# The Relationship between Challenge and Hindrance Stressors and Emotional Exhaustion: The Moderating Role of Perceived Servant Leadership

**DOI:** 10.3390/ijerph17010282

**Published:** 2019-12-31

**Authors:** Hongchao Wu, Shaoping Qiu, Larry M. Dooley, Congying Ma

**Affiliations:** 1 School of Education, South China Normal University, Guangzhou 510631, China; wuhc@scnu.edu.cn (H.W.); congyingma1@gmail.com (C.M.); 2 The Department of Engineering Technology & Industrial Distribution, Texas A& M University, College Station, TX 77843, USA; 3 The Department of Educational Administration & Human Resource Development, Texas A& M University, College Station, TX 77843, USA; l-dooley@tamu.edu

**Keywords:** challenge stressor, hindrance stressor, servant leadership, emotional exhaustion, schoolteachers, China

## Abstract

Schoolteachers worldwide are facing stressful work conditions with heavy responsibilities. Stress may contribute to psychological disorders and physical health issues. The purposes of this study were (1) to investigate whether both challenge and hindrance stressors are positively associated with emotional exhaustion among Chinese schoolteachers and (2) to examine whether perceived servant leadership moderates the effects of challenge and hindrance stressors on emotional exhaustion. This study was cross-sectional in nature. A sample of 2636 schoolteachers was elicited from schools in South China. Research data were collected in the fall semester of 2019 via WeChat^®^. Descriptive statistics and inter-correlations were conducted using SPSS 21. Confirmatory factor analysis was applied to test measurement models to determine convergent and discriminant validities using Mplus 7.4. Hierarchical multiple regression was performed to test proposed hypotheses using SPSS 21. The study results demonstrated that both challenge and hindrance stressors were positively related to emotional exhaustion among schoolteachers in Chinese schools. It was also indicated that, while perceived servant leadership buffers the relationship between challenge stressor and emotional exhaustion, it enhances the relationship between hindrance stressor and emotional exhaustion. Implications and limitations are also provided.

## 1. Introduction

There is a large amount of studies which demonstrate that teaching is among the most stressful of occupations worldwide [1,2,3]. Schoolteachers face mentally and physically stressful work conditions with heavy responsibilities [4]. Although the primary professional duty of a teacher is to interact with and teach students, they also have to contact and meet parents, principals, government officials, and others in order to achieve educational goals. All these parties have their own expectations which, more often than not, are in conflict with each other, resulting in schoolteachers facing multiple challenges and job demands in the course of their teaching career [3]. It has been revealed that most stressors teachers face come from a competitive societal environment, heavy workload, pupil behavior, time demands, work conditions, staff relationships, and family responsibilities [5]. Certain levels and types of job stress are undoubtedly instrumental in triggering teachers’ motivations and generating better teaching performance [6,7]. However, when exceeding organizational and/or personal resources, job stress may have deleterious consequences both to educational objectives and on teachers themselves, ultimately compromising other parties’ interests and well-being [8]. For example, too much stress contributes to serious psychological disorders such as job burnout, anger, frustration, anxiety, depression, and nervousness [5] as well as physical health issues such as headache, dizziness, neck pain, and sleeping problems [9]. In Spain, scholars have revealed that stressors perceived by schoolteachers hamper them from fully meeting learning objectives and cause burnout, anxiety, and depression [10,11]. Particularly, work-related stress was associated with teachers’ engagement and occupational well-being [12]. Similar links between stress and negative psychological outcomes among schoolteachers have been also found in Norway [9,13], USA [14], and South Africa [15]. Stress in the workplace and accruing health problems are especially prevalent among Chinese schoolteachers across all school levels in all parts of China. A research study conducted at 168 schools in China disclosed that the mental and physical stresses schoolteachers suffer are so tremendous that 52.23% of schoolteachers have psychological problems, exemplified by high levels of somatic symptom severity, obsessive-compulsive disorder (OCD), depression, hostility, and fear [16]. It has also been constantly reported in the media that schoolteachers are depressed and emotionally exhausted so much so that they choose to commit suicide and end their lives at very young ages, instead of suffering physical pain and psychological torture resulting from chronic stresses.

Fortunately, the harmful effects of teachers’ physical and psychological health have come to be realized nationally, and major concerns have been raised, especially about early death and suicide. More importantly, significant efforts have been made to reduce the stress that has burdened schoolteachers in China during the last few decades. In spite of these efforts, it appears health issues still remain serious. Behind these detrimental consequences lurk some implicit factors. Aside from the general stressors aforementioned, other social factors unique to China may contribute to these scenarios for Chinese schoolteachers. First, Chinese traditional values place more emphasis on schools and teachers to deliver knowledge to children and adolescents. Children are expected to study diligently to achieve academic excellence to glorify their family [17]. Second, with the continued development of society and deepened education reform, a vast majority of schools in China are adopting the business management model such as performance-based pay without caring more about teachers’ well-being [18]. Third, long-term implementation of the one-child policy in China has resulted in Chinese parents having high expectations for schoolteachers, because they expect their children to achieve academic excellence at an early age [19]. To reiterate, Chinese schoolteachers have to face and tolerate tremendous stresses emanating from the society, government, principals, parents, students, and school policies and procedures.

While stressors manifest themselves in terms of psychological and physiological symptoms and behavioral outcomes, organizational support available to schoolteachers tends to buffer the effect of the stresses on their psychological and physical health [20]. At schools, organizational support includes policies and processes that enhance teachers’ well-being, respecting and trusting school environment, provision of work-related information, mentoring and coaching, supportive communication, leadership, and many others. Servant leadership, as a new leadership paradigm, is one type of social support that holds promise in modern service sectors including education [21]. Greenleaf [22] initially described servant leaders as those who naturally want to serve and to serve first with conscious choice bringing them to aspire to lead. Later, the definition of servant leadership was expanded and redefined as: (1) an other-oriented approach to leadership, (2) manifested through one-on-one prioritizing of follower individual needs and interests (3) and outward reorienting of their concern for self towards concern for others within the organization and the larger community [23].

In this paper, we argue the stressors faced by Chinese schoolteachers is positively linked to their emotional exhaustion. However, the relationship between stressors and emotional exhaustion depends on the level of servant leadership perceived by schoolteachers. We split stressors into two components: challenge stressor and hindrance stressor. The purposes of this study were particularly two-fold. The first was to investigate whether both challenge and hindrance stressors were positively associated with emotional exhaustion. Second, we examined whether perceived servant leadership moderated the effect of challenge and hindrance stressors on emotional exhaustion.

## 2. Theoretical Background and Hypotheses

### 2.1. Theoretical Background

We based our research on job demands–resources (JD–R) model and stress and coping social support theory. The JD–R model posits, while specific job stress factors exist in all occupations, they can be categorized into two general groups: job demands and job resources [24,25]. Job demand refers to physical, psychological, social, or organizational aspects of the job that require sustained physical or mental effort or skills to cope and, therefore, are associated with certain physiological and/or psychological costs [26,27]. Job stressors and job demand are interchangeably used in the literature. Examples are work pressure, role ambiguity, unfavorable working conditions, and problems related to restructuring, emotional demand, and many others [24]. Job resources, on the other hand, point to “physical, psychological, social, or organizational aspects of the job that are either/or: (1) functional in achieving work goals; (2) reduce job demands and the associated physiological and psychological costs; (3) stimulate personal growth and development” [18]. Examples include career opportunities, job security, role clarity, participation in decision making, feedback, autonomy, and a supporting climate [28].

While the JD–R model emphasizes job demands and job resources as two counterbalancing factors associated with exhaustion and depersonalization, stress and coping social support theory is proposed to explicate the mechanism through which stress and health are linked to each other [29]. According to this theory, social support protects individuals from the effects of stressors in terms of psychological and physiological health [30]. Functioning as a stress buffer, social support influences how individuals adaptively appraise the stressors they have to face and accordingly affects the ways individuals can effectively cope with these stressful encounters [31].

### 2.2. The Effects of Challenge and Hindrance Stressors on Emotional Exhaustion

Job stress is formally defined as the subjective evaluation of experienced stress associated with specific job stressors and work outcomes [32,33]. On the basis of its effect on work outcomes, Cavanaugh et al. [32] categorized job stressor into challenge-related stressor and hindrance-related stressor. Challenge stressor is the type that requires effort but benefits an employee’s personal growth and achievement. Factors causing challenge stress include job scope, responsibility, workload, and time pressure. In contrast, hindrance stressor is considered to constrain personal achievement and, thus, hindering an employee’s goal progress. Examples include organizational politics, red tape, job ambiguity, and job insecurity. As a type of burn-out, emotional exhaustion is defined as a state of feeling emotionally overextended and exhausted as a result of accumulated workplace stress [34].

According to the JD–R model, both challenge and hindrance stressors are associated with mental and physical costs including emotional exhaustion. That is to say, the two types of job stressors both have harmful effects on employees’ psychophysiological health to some extent. There is no exception with challenge stressor despite its positive impact on individual’s personal growth and achievement. This reasoning is empirically supported or partially supported by a number of studies. For example, a meta-analytic test by LePine, Podsakoff, and LePine [6] revealed that challenge and hindrance stressors explained 39% of the variance in strain (anxiety, depersonalization, depression, and emotional exhaustion) and both stressors were positively and significantly related to strain with regression weights of 0.23 and 0.50, respectively. Supporting evidence was also found in the studies in the context of education. For instance, Webster, Beehr, and Love [35] found, while challenge appraisal associated with responsibility had a positive relationship with physical symptoms among non-teaching university employees in the USA, hindrance appraisal associated with any stressor was associated with both increased emotional exhaustion and increased physical symptoms. This study implied both hindrance and challenge stressors can have adverse effects on employees’ psychophysiological well-being. Another support comes from the study by Stiglbauer and Zuber [36], who found hindrance stressor was predictive of fatigue, tension, and cardiovascular illnesses among schoolteachers in Austria. However, challenge stress was not found to be significantly related to emotional exhaustion among schoolteachers in their study.

On the basis of the above theories and literature, we expected hindrance stressor would positively correlate with emotional exhaustion among Chinese schoolteachers. Along similar lines, we proposed challenge stressor would also positively associate with emotional exhaustion among Chinese schoolteachers, despite the fact that the study results were mixed for the relationship between challenge stressor and emotional exhaustion. The reasons are, just like for hindrance stressor, challenge stressor was also associated with physical, psychological, social, or organizational aspects of the job that required sustained physical or mental effort or skills to cope with and, therefore, was closely related to certain physiological and/or psychological costs [26,27]. Hence, we proposed two hypotheses:

**Hypothesis** **1** **(H1).** *Challenge stressors are positively related to emotional exhaustion among schoolteachers in Chinese schools.*

**Hypothesis** **2** **(H2).** *Hindrance stressors are positively related to emotional exhaustion among schoolteachers in Chinese schools.*

### 2.3. Moderating Role of Perceived Servant Leadership

As illustrated previously, stress and coping social support theory posited that social support affects individuals’ adaptive assessment of stresses, providing a coping strategy for employees to handle the stressful encounters. Thus, social support can be an effective moderator impacting the relationship between stressors and emotional exhaustion [37]. As one type of social support, servant leadership protects schoolteachers from the effects of stressors in terms of psychological and physiological health in various ways. First, servant leaders exhibit empathy and compassion and heal teacher’s emotional sufferings [38]. Second, they define and create a safe and trusting work environment for teachers to work [39]. Third, they craft a compelling school vision [40] and empower teachers to become autonomous in order to develop their talent [41]. Last, servant leaders listen to teachers’ voice and enhance communication among teachers and leaders [42]. Empirical evidence demonstrated the positive relationship between servant leadership and employees’ psychological health [43]. Following the same reasoning, we argued servant leadership also functions as a moderator between stressors and emotional exhaustion. For those who perceive a high level of servant leadership from their leaders, servant leadership would buffer these relationships. For those with a perceived low servant leadership, such relationships would be enhanced. Therefore, we proposed:

**Hypothesis** **3** **(H3).** *Perceived servant leadership moderates the relationship between two stressors and emotional exhaustion. In particular, the positive relationship between two stressors and emotional exhaustion is weaker for schoolteachers who have a high level of perceived servant leadership (buffer effect).*

**Figure 1:**
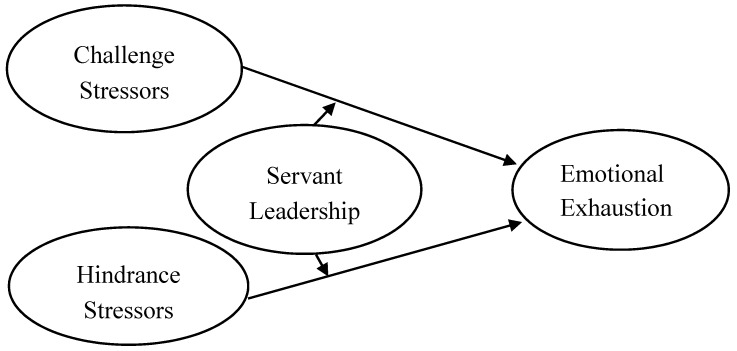
Hypothesized conceptual model.

Based on the above hypotheses, we created a conceptual model (see Figure 1).

## 3. Materials and Methods

### 3.1. Participants and Procedures

The study design was cross-sectional in nature. The instruments were originally developed and used in Western countries. Back-translations were performed by two Chinese professors to guarantee accuracy in meaning and cultural suitability. All surveys were administered in Chinese. A non-probability sample of 3200 schoolteachers was elicited from schools in South China. Research data were collected in the fall semester of 2019 through self-report questionnaires. We choose this sampling technique because a class of students of the first author were taking internships at schools of various education level across different regions in South China. The student teachers could reach out to contact participants. After obtaining informed consent, the student teachers sent a survey link to participants through WeChat^®^. A week later, a reminder text was sent to ask them to complete the survey. To minimize common method bias (CMB), anonymity and confidentiality of the participants were ensured, clear instructions for completing the measures were provided, and complicated wording was avoided in translating the instrument scales [44]. In addition, items from 4 variables were intermixed to further reduce common method variance [45]. Prior to data collection, a pilot study was conducted using 25 schoolteachers. Based on their feedbacks, necessary alterations were made.

Of 3200 schoolteachers we initially contacted, a total of 2636 participants returned the survey with a response rate of 82.4%; all teachers provided valid data. Seven hundred and seven participants (26.8%) were men while 1929 (73.2%) were women. The mean age of participating teachers was 36.49 years (SD = 9.03). There were 2220 public schoolteachers (84.2%) and 416 private school participants (15.8%). A total of 1272 (48.3%) participants were from elementary schools, whereas 506 (19.2%) were middle schoolteachers and 858 (32.5%) teachers came from high schools. The average teaching tenure was 15.36 years (SD = 55.56), and the participating teachers earned 88.2 thousand (SD = 58,830) CNYs per year on average. Table 1 presents the descriptive characteristics of the sample.

### 3.2. Measures

Participants responded to each item of the constructs using a 5-point Likert-type scale ranging from 1 “strongly disagree” to 5 “strongly agree”. A high score participants rated on an item represented a high level on the corresponding construct.

#### 3.2.1. Stressors

Challenge stressors and hindrance stressors were measured using a stressor measurement scale developed by LePine, Zhang, Crawford, and Rich [46]. The measurement scale consisted of 10 challenge-related items and 10 hindrance-related items. An example item of a challenge stressor measure included “I have to work at a rapid pace to complete all of my tasks”. An example item of a hindrance stressor measure was “I often receive conflicting instructions and expectations from my boss or bosses”. The reported consistency reliabilities were 0.90 and 0.93 for the challenge measure and the hindrance measure, respectively [46]. Our current study found a reliability estimate of 0.88 for both the challenge and hindrance stressor measures.

#### 3.2.2. Perceived Servant Leadership

Perceived servant leadership was assessed using the 6-item Short Form of the Servant Leadership Behavior Scale (SLBS-6) developed by Sendjaya, Eva, Butar, Robin, and Castles [47]. Participants rated the extent to which they perceived their principals to display leadership behaviors on the six items. An example item was “My principal uses power in service to others, not for his or her ambition”. The Cronbach’s α reported in a previous study was 0.89 [47]. The present study found a consistency reliability of 0.82 for this construct.

#### 3.2.3. Emotional Exhaustion

Emotional exhaustion was measured by means of a 9-item emotional exhaustion subscale of the Maslach Burnout Inventory (MBI) [48]. The participants rated the degree to which they felt emotionally overextended or exhausted by their work. An example item included was “I feel emotionally drained from my work”. In their study, Maslach and Jackson [48] reported an estimated Cronbach’s α to be 0.89. The consistency reliability of current study was 0.90.

#### 3.2.4. Control Variables

Control variables in this study included gender, age, annual income, teaching tenure, school level, and school type. Prior research has found these variables are related to teachers’ physical and psychological well-being [3,49,50,51].

### 3.3. Analytical Strategies

First, descriptive statistics such as mean, standard deviation, skewness, and kurtosis were computed to examine the central tendency, dispersion, and distribution. Zero-order correlations were subsequently calculated to determine the magnitudes of the associations among variables. Additionally, variance inflation factor (VIF) was also detected to diagnose multicollinearity. Consistency reliabilities of the variables were evaluated by means of computing Cronbach’s α. All these analyses were conducted using the SPSS statistical software (version 21). Then, confirmatory factor analysis (CFA) was applied to test measurement models to determine convergent and discriminant validities. Fit indices used to determine model fit were chi-square (*x*^2^), root mean square error of approximation (RMSEA), standardized root mean square residual (SRMR), Comparative Fit Index (CFI), and the Tucker–Lewis Index (TLI). The statistical software Mplus 7.4 was used to conduct CFA. Finally, hierarchical multiple regression was performed to test the proposed hypotheses using SPSS 21.

## 4. Results

### 4.1. Descriptive Statistics, Reliabilities, and Intercorrelations

We performed data statistics analysis on all the variables including means, standard deviations, reliability coefficient, and correlation coefficient among all the research variables. The results of the analysis are displayed in Table 2. It can be seen that emotional exhaustion was highly positively correlated with both challenge stressor (*r* = 0.63, *p* < 0.01) and hindrance stressor (*r* = 0.74, *p* < 0.01). However, the relationship between emotional exhaustion and perceived servant leadership was negative, though still significant. Therefore, H1 and H2 were initially supported. While the challenge and hindrance stressors were positively associated with each other (*r* = 0.60, *p* < 0.01), these two stressors were negatively correlated with perceived servant leadership. It was also shown, of all the six control variables, only age, school level, and school type were significantly related to emotional exhaustion.

### 4.2. Normal Distribution, Collinearity, and Common Methods Bias

After collecting the data, we performed data screening. There were no missing data or outliers in the data. The skewness indices of all variable items were within the range of −1 and +1, whereas the kurtosis indices were within −3 and +3 [52]. Therefore, the data distributions could be considered close to normal distribution. The variance inflation factors (VIFs) scores were close to 1, indicating no major collinearity issues. Although we used several procedural remedy techniques to control for the CMB, we still checked whether there were CMB issues in the data. The result of Harman’s single factor test indicated one single factor accounted for 33.96% of the variance, much lower than 50%, indicating no major issues with CMB.

### 4.3. Convergent Validity and Discriminant Validity

The CFA analysis was conducted to examine the convergent validity and discriminant validity of the main variables (i.e., challenge stressor, hindrance stressor, perceived servant leadership, and emotional exhaustion). To this end, we compared four measurement models for the main variables. While challenge stressor and hindrance stressor were combined as one factor in the three-factor model, two-factor model combined two stressors and perceived servant leadership. The fit indices of four models are provided in Table 3. The results indicated the four-factor model fit the data better than any other models (χ^2^ = 3192.95, *df* = 293, RMSEA = 0.06, CFI = 0.90, TLI = 0.90, SRMR = 0.05). Therefore, the discriminant validity was achieved in this study. Furthermore, in the four-factor model, all the factor loadings for the items were greater than 0.50, and all the values of average variance extracted (AVE) for the four factors were also greater than 0.50, indicating all the four variables had convergent validity.

### 4.4. Hypothesis Testing

We performed hierarchical multiple regression analysis to test the proposed hypotheses using SPSS 21. Since gender, tenure, and annual income were not significantly associated with emotional exhaustion, we excluded these three control variables into the hierarchical linear regression models. We entered three control variables (i.e., age, school level, and school type), three independent variables (i.e., challenge stress, hindrance stress, and servant leadership), and interactions between two stressors and perceived servant leadership into the model in separate steps. The results were displayed in Table 4. In Hypothesis 1 and 2, we posited that both challenge and hindrance stressors are positively related to emotional exhaustion among schoolteachers in Chinese schools. As can be seen from Table 4, the standardized regression coefficients from challenge stressor to emotional exhaustion in Model 2 and Model 3 were positively significant (*β* = 0.30, *p* < 0.01; *β* = 0.32, *p* < 0.01, respectively). Therefore, H1 was supported. In addition, standardized regression coefficients from hindrance stressor to emotional exhaustion in Model 2 and Model 3 were also positively significant (*β* = 0.52, *p* < 0.01 and *β* = 0.50, *p* < 0.01, respectively). Thus, H2 was also confirmed. Last, the standardized regression coefficients for two interactions were all statistically significant (*β* = −0.04, *p* < 0.05 and *β* = 0.07, *p* < 0.01, respectively) as can be seen in Model 3. Perceived servant leadership moderated the relationship between two stressors and emotional exhaustion.

**Figure 2:**
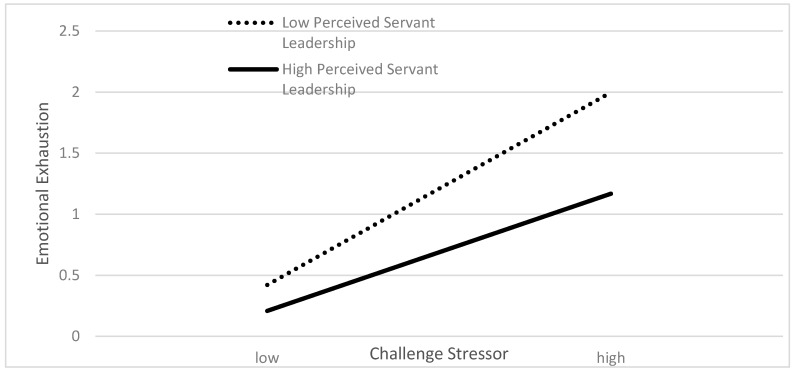
Moderating effect of perceived servant leadership on the relationship between challenge stressor and emotional exhaustion.

**Figure 3:**
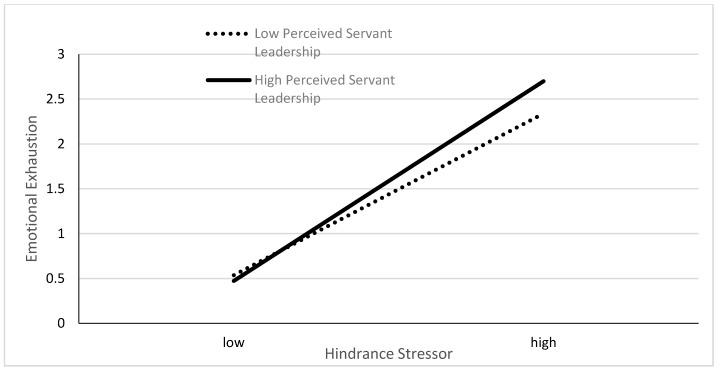
Moderating effect of perceived servant leadership on the relationship between challenge stressor and emotional exhaustion.

To further depict the interaction effects, we followed the recommendations made by Aiken and West [53] and decomposed the interaction terms by splitting perceived servant leadership into two groups: high perceived servant leadership (+1 standard deviation) and low perceived servant leadership (−1 standard deviation). Figure 2 and Figure 3 illustrate the moderating effects of perceived servant leadership on the relationships between two stressors and emotional exhaustion.

As indicated in Figure 2, for both high and low perceived servant leadership groups, a change in challenge stressor upon schoolteachers had a significant positive association with a change in emotional exhaustion. However, the positive influence of the challenge stressor was weaker in the high perceived servant leadership group than the low group. This simply implies perceived servant leadership buffered the relationship between challenge stressor and emotional exhaustion as expected. Similarly, Figure 3 also showed, for both high and low perceived servant leadership groups, that a change in hindrance stressor upon schoolteachers had a significant positive association with a change in emotional exhaustion. Nevertheless, the positive influence of the hindrance stressor was stronger in the high perceived servant leadership group than the low group. This indicated that perceived servant leadership enhanced the relationship between challenge stressor and emotional exhaustion which was contrary to our expectations. Therefore, hypothesis 3 was partially supported.

## 5. Discussion

Using a sample of 2636 teachers recruited from schools in the southern part of China, we examined how challenge and hindrance stressors influence emotional exhaustion among schoolteachers. The moderating effects of perceived servant leadership were also tested between these two stressors and emotional exhaustion. The hypotheses we proposed were largely supported.

First, the results of this study clearly established both challenge and hindrance stressors are positively associated with emotional exhaustion among Chinese schoolteachers. The more challenge and hindrance stressors imposed on teachers, the more severe emotional exhaustion they have to confront. These findings are in agreement with the JD–R model which posits all job stressors are associated with mental and physical costs, such as emotional exhaustion; to a certain degree. Both challenge and hindrance stressors have deleterious effects on schoolteachers’ psychophysiological health. Similar results were also reported by LePine, Podsakoff, and LePine [6] and Webster, Beehr, and Love [35], each of the studies found that both challenge and hindrance stressors can predict schoolteachers’ physical and psychological well-being. Given this positive relationship, Chinese government has recently taken measures to lessen schoolteachers’ stresses in an attempt to improve their physical and psychological health. These measures include reducing teachers’ workload, streamlining administrative procedures to reduce administrative hassles, increasing more resources to accomplish tasks, and providing psychological counselling. All these initiatives might be applicable to different cultures and other countries.

Second, this study revealed schoolteachers’ perceived servant leadership buffers the relationship between challenge stressor and exhaustion. In other words, positive correlation of the challenge stressor with emotional exhaustion is weaker for those with high level of perceived servant leadership versus low level. This finding dovetails with stress and coping social support theory. Servant leaders provide emotional healing and other supports to help employees grow [54]. In a sense, servant leadership functions as layers of cushion to protect schoolteachers from harmful impact of challenge stressor. With this in mind, Chinese school leaders are now switching their once-effective business model to more humanized management paradigm, providing more physical, emotional, and social support to schoolteachers. This way, Chinese schoolteachers benefit from lowered occurrence or reduced severity of psychophysiological health issues even though they have to face heavy challenge stressors.

Third, it was disclosed schoolteachers’ perceived servant leadership moderates the relationship between hindrance stressor and emotional exhaustion. To our surprise, however, we failed to find the attenuating effect of perceived servant leadership. Instead, perceived servant leadership enhances the harmful impact of hindrance stressor on emotional exhaustion, particularly for schoolteachers who undergo high level of hindrance stressor. Several reasons might explain why high level of hindrance stressor combined with high level of perceived servant leadership would exacerbate schoolteachers’ health. First, although controlled for gender, age, income, school level, and other variables in this study, we might ignore other implicit or unnoticed yet important variables that affect schoolteachers’ psychological health, or we neglect some intermediate variables that might exert influence on the relationship between stressors and emotional exhaustion. Second, the “black box” connecting hindrance stressor and emotional exhaustion might be more complex than expected and might not be explained by linear regression. Their relationship could rather be curvilinear or exponential. Using linear regression to explain can only oversimplify the whole moderating mechanism. Third, cognitive dissonance theory [55] proposes individuals’ cognitive dissonance usually results from holding two or more conflicting perceptions simultaneously, leading to psychological stress. On the one hand, servant leaders display altruistic behaviors and provide emotional support to schoolteachers [56]. On the other hand, schoolteachers have to suffer from high level of hindrance stressors, such as administrative hassles, conflicting instructions, bureaucratic constraints, and unclear job tasks. In such a school culture, teachers most probably attribute high level stress to their leaders because school leaders design and implement policies and rules. For schoolteachers undergoing high stress, leaders are the sources of these hindrance stressor and thus the culprit of their sufferings. It is leaders who impose the hindrance stressor on them, such that teachers perceive their leaders’ altruistic behaviors as inauthentic or fake, contributing to their cognitive dissonance. Coupled with new psychological stress caused by this cognitive dissonance, schoolteachers suffering high level of hindrance stress might produce more discomfort and emotional exhaustion. For example, dispute with peers and conflicting instructions from school leaders are two sources of hindrance stressor. In a school where teachers frequently dispute with each other, school leaders have to resolve the conflicts when occurring. However, providing emotional healing to schoolteachers might simply add fuel to the flames, making bad situations worse. It was reported that schoolteachers in this situation backfired and targeted their anger at their leaders because they interpreted leaders’ behaviors as inauthentic, leading to more distress and even physical illnesses.

## 6. Implications

### 6.1. Theoretical Implications

This study makes several contributions to leadership and employees’ psychological health literature. First, although workplace health has become more important nowadays, there is still scant research available in the literature focusing on the relationship between servant leadership and employees’ psychological health [44,57,58]. Recent systematic literature review studies on servant leadership did not even take psychological health into consideration as an outcome construct [23,59]. Therefore, the current study adds to the literature, enhancing our understanding of the association of servant leadership with employees’ well-being.

Second, our study confirmed the JD–R model by demonstrating challenge and hindrance stressors are both associated with mental and physical costs including emotional exhaustion. However, running counter to our intuitive and expectations, we found servant leadership does not dampen the relationship between hindrance stressor and emotional exhaustion among Chinese schoolteachers. This might provoke interested researchers to replicate the study in other cultures or other industries or at least conduct similar studies using the relevant variables to confirm or overthrow the hypothesized relationships.

### 6.2. Practical Implication

The current study also has some practical implications. Firstly, the results of our study indicated both challenge and hindrance stressors were positively related to schoolteachers’ emotional exhaustion. Apparently, school principals and other leaders need to decrease the stressors burdened on the shoulders of teachers as much as possible in order to lessen the stresses and minimize the likelihood of the occurrence of physical and psychological illness. Secondly, although not hypothesized, perceived servant leadership was found to be negatively associated with emotional exhaustion. Additionally, perceived servant leadership can buffer the relationship between challenge stressor and emotional exhaustion. It appears school governments who seek to improve teachers’ psychological health need to encourage their leaders to wholeheartedly display servant leadership behaviors on a daily basis. Education authorities could conduct leadership training programs to train current school leaders. Finally, servant leadership is a double-edged sword. School leaders should be cautious that, for schoolteachers with high-level hindrance stressors, showing servant leadership could worsen the situation, because it would increase the likelihood of teachers being emotionally exhausted. In this case, it would be a better strategy if school leaders turned back to the sources of the stress and reduced teachers’ burden. Instead of guessing, leaders should perform a job–task analysis on the teachers in question to determine the tasks they are performing. To assume the leader knows for sure how a teacher spends their time is a fallacy. Additionally, school leaders can fairly resolve disputes and conflicts occurring among the teachers, from the analysis above, give teachers clear instructions and job tasks, reduce administrative hassles, and build a fair reward and promotion system.

### 6.3. Limitations and Future Research

There are several limitations in this study. First and foremost, servant leadership is basically a group level construct. However, we did not adopt a hierarchical linear model (HLM) to analyze the sample data. Instead, we used perceived servant leadership as a construct to measure schoolteachers’ perception on individual level. Future research is encouraged to utilize servant leadership as a group level construct to capture school leaders’ behaviors associated with servant leadership. Next, as aforementioned, we might ignore some important control variables or moderating variables in this study. Scholars investigating similar relationships could identify such variables to derive more in-depth conclusions in regards to the association of stress with resultant psychological well-being. Third, we collected data from schoolteachers in southern part of China using snowball sampling technique. The sample may not represent the whole population of schoolteachers in China. For example, in the current study, only 26% of the participants were male teachers and 15% worked in private schools. Researchers should be circumspect when they generalize the study results or replicate the study to other regions of China, other industries, or other cultures. Lastly, this study was cross-sectional in nature. Therefore, we tried not to use any causation-induced words to describe the relationships. To test whether stressors cause emotional exhaustion, future research should use a longitudinal study or experimental design to strictly control for other conditions or confounding variables.

## 7. Conclusions

In this study, we examined how challenge and hindrance stressors influence emotional exhaustion among schoolteachers in China. We also tested the moderating effects of perceived servant leadership between these two stressors and emotional exhaustion. The study results indicated both challenge and hindrance stressors are positively related to schoolteachers’ emotional exhaustion. It is also revealed that, while perceived servant leadership buffers the relationship between challenge stressor and schoolteachers’ emotional exhaustion, it enhances the association between hindrance stressor and emotional exhaustion. For those who perceive a high level of servant leadership from their school leaders, unhealthy emotional exhaustion is more likely to occur when teachers shoulder a high level of hindrance stressors.

## Figures and Tables

**Table 1:** Descriptive characteristics of the sample.

Description	Percent (%)
**Gender**	
Men	26.8
Women	73.2
**Age**	
20s	32.1
30s	36.7
40s	25.1
Above 50s	6.1
**Teaching tenure**	
1–10 years	44.4
11–20 years	29.5
21–30 years	21.2
Above 30 years	4.9
**School level**	
Elementary school	48.3
Middle school	19.2
High school	32.5
**School type**	
Public school	84.2
Private school	15.8
**Annual income (Thousand CNY)**	
Below 50	31.5
50–99	43.1
100–149	15.3
Above 149	10.1

**Table 2:** Means, standard deviations, reliabilities, and intercorrelations among variables.

Variable	Mean	SD	1	2	3	4	5	6	7	8	9	10
1. Gender	1.73	0.44										
2. Age	36.49	9.03	−0.27									
3. Tenure	15.36	55.56	−0.03	0.14 **								
4. School level	1.84	0.89	−0.17 **	0.13 **	0.03							
5. School type	1.16	0.37	0.04 *	−0.16 **	−0.00	−0.20 **						
6. Income	8.82	5.88	−0.07 **	0.24 **	0.05 *	0.10 **	−0.03					
7. CS	3.75	0.67	−0.03	−0.00	−0.02	−0.09 **	−0.03	0.12 **	0.88			
8. HS	3.01	0.73	−0.13	0.04	−0.02	0.08 **	−0.11 **	0.08 **	0.60 **	0.88		
9. SL	3.04	0.77	0.03	−0.03	0.02	−0.17 **	0.06 **	0.00	−0.09 **	−0.44 **	0.82	
10. EE	2.65	0.76	−0.03	−0.04 *	−0.01	−0.07 **	−0.07 **	−0.02	0.63 **	0.74 **	−0.34 **	0.90

Note: ** *p* < 0.01, * *p* < 0.05. CS represents challenge stress; HS denotes hindrance stress; SL is perceived servant leadership; EE denotes emotional exhaustion; SD is standard deviation. The reliability coefficient is in the diagonal.

**Table 3:** Model comparison.

Model	χ^2^	*df*	Δχ^2^	RMSEA	CFI	TLI	SRMR
**Four-factor model** **CS, HS, SL, EE**	3192.95	293		0.06	0.90	0.90	0.05
**Three-factor model** **CS + HS, SL, EE**	4871.60	296	1678.65 **	0.08	0.85	0.83	0.07
**Two-factor model** **CS + HS + SL, EE**	5873.65	298	2680.70 **	0.08	0.81	0.79	0.07
**One factor model** **CS + HS + SL+ EE**	8595.85	299	5402.90 **	0.10	0.72	0.69	0.09

Note: CS denotes challenge stress; HS represents hindrance stress; SL indicates perceived servant leadership; EE is emotional exhaustion; RMSEA: root mean square error of approximation; SRMR: standardized root mean square residual; CFI: comparative Fit Index; TLI: the Tucker–Lewis Index; ** *p* < 0.01.

**Table 4:** Results of hierarchical multiple regression for hypotheses testing.

Emotional Exhaustion			
Variable	Model 1	Model 2	Model 3
*Control Variables*			
Age	−0.05 *	−0.06 **	−0.06 **
School level	−0.08 **	−0.10 **	−0.10 **
School type	−0.10 **	−0.03 *	−0.03 *
*Predictors*			
CS		30 **	0.32 **
HS		0.52 **	0.50 **
SL		−0.11 **	−0.12 **
CS × SL			−0.04 *
HS × SL			0.07 **
*R* ^2^	0.01 **	0.62 **	0.62 **
F	12.69 **	718.71 **	544.81 **

Note: CS denotes challenge stress; HS represents hindrance stress; SL indicates servant leadership; EE is emotional exhaustion; ** *p* < 0.01; * *p* < 0.05.
